# Blunted Lsm14b activation disrupts maternal mRNA remodeling during the GV-to-MII transition in a mouse model of polycystic ovary syndrome

**DOI:** 10.3389/fcell.2026.1889968

**Published:** 2026-07-30

**Authors:** Wenchen Du, Jiayu Shi, Jie Chen, Mengyu Zhang, Xiaojie Jiang, Ruxin Wang, Yong Tan

**Affiliations:** 1 First College of Clinical Medicine, Nanjing University of Chinese Medicine, Nanjing, Jiangsu, China; 2 Medical Experimental Research Center, First College of Clinical Medicine, Nanjing University of Chinese Medicine, Nanjing, Jiangsu, China; 3 Affiliated Hospital of Nanjing University of Chinese Medicine, Nanjing, Jiangsu, China

**Keywords:** GV-to-MII transition, *Lsm14b*, maternal mRNA clearance, oocyte maturation, polycystic ovary syndrome, processing body, transcriptomics

## Abstract

**Introduction:**

Polycystic ovary syndrome (PCOS) is the leading cause of anovulatory infertility and is consistently associated with poor oocyte developmental competence, yet the molecular basis of this qualitative defect remains poorly defined. Because the germinal vesicle (GV)-to-metaphase II (MII) transition occurs under global transcriptional silence and depends entirely on the post-transcriptional remodeling of pre-stored maternal mRNAs, we hypothesized that PCOS may selectively disrupt this regulatory program rather than impose a constitutive transcriptomic lesion.

**Methods:**

To test this, we established a DHEA-induced PCOS mouse model, validated by estrous acyclicity, hyperandrogenism, and polycystic ovarian morphology, and performed parallel Smart-seq2 profiling of oocytes at both the GV and MII stages. Integrated analyses combining differential expression, maturation trajectory modeling, weighted gene co-expression network analysis (WGCNA), and protein–protein interaction mapping, together with independent RT-qPCR validation of four hub transcripts, were used to dissect stage-specific transcriptomic dynamics.

**Results:**

Control and PCOS oocytes were largely similar at the single-gene level at the GV stage (35 differentially expressed genes), with subtle network-level perturbations detectable only by co-expression analysis, but diverged dramatically upon meiotic maturation (292 differentially expressed genes), with the majority of dynamic transcripts diverted into pathological trajectories. This maturation-coupled collapse manifested as a dual-layered post-transcriptional failure: aberrant retention of maternal mitochondrial OXPHOS transcripts (*Sdhb, Cox6a1*) reflecting impaired mRNA clearance, and concurrent hyper-depletion of oocyte identity genes (*Figla, Zp3*). Mechanistically, Lsm14b, an essential P-body assembly factor, failed to execute its physiological upregulation during the GV-to-MII transition in PCOS oocytes (1.49-fold induction in PCOS versus 9.63-fold in controls; an ∼6.5-fold reduction), providing a proximal explanation for the global decay-machinery failure. RT-qPCR independently confirmed stage-specific dysregulation, including ∼34-fold excessive depletion of *Figla* exclusively at the MII stage. Western blot analysis of Lsm14b, Sdhb, and Figla proteins in GV and MII oocytes confirmed that all three key transcriptomic findings are recapitulated at the protein level, establishing that the mRNA-level dysregulation has functional consequences for protein output.

**Discussion:**

These findings reframe PCOS-associatedoocyte dysfunction as a stage-specific failure of post-transcriptional remodeling competence and identify the Lsm14b–P-body axis as a candidate molecular target for improving oocyte quality in assisted reproduction.

## 1 Introduction

Polycystic ovary syndrome (PCOS) is the most prevalent endocrine disorder in women of reproductive age, affecting 8%–13% of the global female population and representing the leading cause of anovulatory infertility (Almhmoud et al., 2024; Joham et al., 2022).Clinically, PCOS is defined by the Rotterdam criteria as the presence of at least two of three features: hyperandrogenism, oligo- or anovulation, and polycystic ovarian morphology, frequently accompanied by insulin resistance and metabolic comorbidities (Choudhari et al., 2024). Despite its high prevalence, the molecular mechanisms driving the associated reproductive failure remain incompletely understood. A particularly vexing clinical paradox is that PCOS patients undergoing controlled ovarian stimulation typically yield a greater number of oocytes than non-PCOS controls, yet consistently achieve lower fertilization rates, poorer embryo quality, and reduced live birth rates in assisted reproductive technology (ART) cycles (Andreeva et al., 2023; Jain et al., 2022; Sayutti et al., 2022; Turathum et al., 2021). Current clinical interventions for PCOS-associated infertility, including ovarian stimulation and *in vitro* maturation (IVM) protocols, primarily address the quantitative dimension of this paradox; the molecular basis of the qualitative oocyte defect has remained a critical unresolved question. This dichotomy—quantitative oocyte abundance coexisting with qualitative incompetence—points to intrinsic molecular defects within the PCOS oocyte itself.

The acquisition of oocyte developmental competence relies critically on the precise spatiotemporal regulation of maternal mRNA dynamics. In fully grown oocytes, transcription is globally silenced from the late germinal vesicle (GV) stage onward and only resumes upon embryonic genome activation (EGA) following fertilization (Christou-Kent et al., 2020; Jiang et al., 2023). Consequently, the oocyte becomes entirely dependent on the orchestrated storage, translational activation, and programmed clearance of pre-existing maternal mRNAs to successfully navigate the GV-to-MII transition and support early embryogenesis (Sha et al., 2020). This post-transcriptional remodeling program is coordinated by a network of RNA-binding proteins (RBPs) and ribonucleoprotein granules, including processing bodies (P-bodies)—cytoplasmic ribonucleoprotein condensates that serve as primary hubs for maternal mRNA storage, translational repression, and programmed decay during meiotic maturation (Standart and Weil, 2018; Sha et al., 2018). A central component of this machinery is Lsm14b, an oocyte-enriched RBP that nucleates P-body assembly and is indispensable for the timely storage and clearance of maternal transcripts; its genetic ablation in mice causes meiotic arrest and female infertility (Wan et al., 2023; Li et al., 2023; Shan et al., 2023; Zhang et al., 2024). Disruption of this carefully timed RNA regulatory program invariably results in reproductive failure (Rong et al., 2019; Sha et al., 2018; Zhao et al., 2020). However, the extent to which PCOS pathology intersects with this fundamental post-transcriptional regulatory axis has not been systematically investigated.

Prior transcriptomic investigations have provided valuable insights into the molecular landscape of PCOS oocytes, identifying dysregulated pathways related to mitochondrial function, cell cycle control, and oxidative stress (Danfeng et al., 2023; Qi et al., 2020). Nevertheless, these studies share critical limitations. They have predominantly profiled oocytes at a single developmental timepoint, precluding the identification of stage-specific transcriptomic transitions. For instance, while single-cell approaches have profiled clinical PCOS oocytes at the MII stage and revealed mitochondrial dynamics (Qi et al., 2020), they lack systematic trajectory analyses of the maturation-coupled transcriptomic remodeling program. Similarly, studies employing mouse models have largely examined oocytes at a single stage rather than capturing the full GV-to-MII transition (Danfeng et al., 2023). This static approach obscures the precise developmental window at which PCOS-induced dysregulation manifests. Consequently, whether PCOS imposes a constitutive transcriptomic defect present from the GV stage, or whether it selectively derails the dynamic remodeling that occurs during meiotic resumption, represents a clinically vital knowledge gap.

To address these gaps, we performed parallel Smart-seq2 transcriptomic profiling of oocytes collected at both the GV and MII stages from a well-validated DHEA-induced PCOS mouse model, enabling a high-resolution temporal characterization of transcriptomic dynamics. Through integrated multi-layered analyses—encompassing trajectory modeling, weighted gene co-expression network analysis (WGCNA), and interaction mapping—we reveal that PCOS oocytes maintain an apparent transcriptomic parity at the GV stage, but undergo a profound and selective transcriptomic collapse specifically during the GV-to-MII transition. This maturation-coupled crisis is characterized by the blunted activation of Lsm14b, a central P-body assembly factor, and manifests as a dual-layered post-transcriptional catastrophe: the pathological retention of metabolic transcripts (*Sdhb, Cox6a1*) and the aberrant hyper-depletion of oocyte identity genes (*Figla*). Supported by independent RT-qPCR validation, these findings define a previously unrecognized transcriptomic vulnerability window in PCOS oocytes, offering new mechanistic insights into the impaired developmental competence observed in clinical settings.

## 2 Materials and methods

### 2.1 Animals and ethics statement

Four-week-old female ICR (Institute of Cancer Research) mice were purchased from Beijing Vital River Laboratory Animal Technology Co., Ltd. [SYXK (Jing) 2023-0077] and housed in the Specific Pathogen-Free (SPF) barrier system at the Experimental Animal Center of Nanjing University of Chinese Medicine. The mice were maintained under standard laboratory conditions: a temperature of 22 °C–24 °C, a relative humidity of 55%–70%, and a 12-h light/dark cycle, with food and water provided *ad libitum*. All animal experiments were reviewed and approved by the Experimental Animal Ethics Committee of Nanjing University of Chinese Medicine (No. 202506A082).

### 2.2 DHEA-induced PCOS mouse model and oocyte collection

The PCOS model was established by subcutaneous injection of dehydroepiandrosterone (DHEA; 6 mg/100 g body weight, dissolved in 0.1 mL sesame oil) once daily for 21 consecutive days, beginning at 4 weeks of age (Motta, 2010; Xu et al., 2021). Control mice received an equivalent volume of the sesame oil vehicle. To collect germinal vesicle (GV)-stage oocytes, mice were injected intraperitoneally with 10 IU pregnant mare’s serum gonadotropin (PMSG) 44–48 h prior to collection. Ovaries were dissected and placed in M2 medium supplemented with 2.5 μM milrinone to prevent spontaneous meiotic resumption. Cumulus–oocyte complexes (COCs) were released by puncturing antral follicles with a 27-gauge needle, and denuded GV oocytes were obtained by brief mechanical pipetting following hyaluronidase treatment. To collect metaphase II (MII)-stage oocytes, mice were injected with 10 IU human chorionic gonadotropin (hCG) 14–16 h after PMSG priming. Oviducts were dissected 14–16 h post-hCG, and COCs were released into M2 medium. MII oocytes were denuded using hyaluronidase and morphologically confirmed by the presence of the first polar body.

### 2.3 Estrous cycle monitoring

Estrous cycle stages were monitored daily by vaginal cytology during days 12–21 of DHEA or vehicle administration. Briefly, a small volume of sterile phosphate-buffered saline (PBS) was flushed into the vaginal canal using a blunt-tipped pipette, and the lavage was immediately transferred onto a glass slide. After air-drying, slides were stained with crystal violet and examined under light microscopy. Cycle stages were classified as proestrus (P), estrus (E), metestrus (M), or diestrus (D) based on the predominant cell type observed: nucleated epithelial cells (proestrus), cornified anucleated cells (estrus), mixed leukocytes and epithelial cells (metestrus), and predominantly leukocytes (diestrus).

### 2.4 Serum hormone assays

Blood samples were collected by retro-orbital puncture under isoflurane anesthesia at the end of the modeling period. Serum was separated by centrifugation at 3,000 × *g* for 15 min at 4 °C and stored at −80 °C until analysis. Serum concentrations of testosterone (T), estradiol (E2), follicle-stimulating hormone (FSH), and luteinizing hormone (LH) were measured using commercially available enzyme-linked immunosorbent assay (ELISA) kits (CUSABIO Technology LLC, Wuhan, China): Mouse Testosterone ELISA Kit (Cat. No. CSB-E05101m, Lot No. J08015416), Mouse Estradiol (E2) ELISA Kit (Cat. No. CSB-E05109m, Lot No. C0154050128), Mouse LH ELISA Kit (Cat. No. CSB-E12770m, Lot No. C0154020125), and Mouse FSH ELISA Kit (Cat. No. CSB-E06871m, Lot No. C0154040127). All samples were measured in duplicate, and the mean value was used for statistical analysis. All procedures were carried out according to the kit instructions.

### 2.5 Ovarian histology and follicle counting

Ovaries were dissected, fixed in 4% paraformaldehyde (PFA) overnight at 4 °C, dehydrated through a graded ethanol series, cleared in xylene, and embedded in paraffin. Serial sections of 5 μm thickness were cut throughout the entire ovary at 30 μm intervals and stained with hematoxylin and eosin (H&E). Follicle classification and counting were performed on every fifth section under light microscopy by two independent observers blinded to the experimental groups. Follicles were classified as: primordial (oocyte surrounded by a single layer of flattened granulosa cells), primary (oocyte surrounded by a single layer of cuboidal granulosa cells), secondary (oocyte surrounded by two or more layers of granulosa cells without an antrum), and antral (follicles with a visible fluid-filled antrum). Cystic follicles were defined as large follicles with a thin granulosa cell layer and an expanded antrum. Corpora lutea were identified by their characteristic luteinized cell morphology. Total follicle numbers per ovary were estimated using the Abercrombie correction formula (Abercrombie, 1946).

### 2.6 Smart-seq2 library preparation and sequencing

For transcriptomic profiling, pools of 10 morphologically normal oocytes per sample were collected directly into lysis buffer on ice. Full-length cDNA was synthesized and amplified using the SMART-Seq v4 Ultra Low Input RNA Kit (Clontech/Takara Bio, Cat. No. 634888). Briefly, mRNA was captured via oligo-dT priming, reverse-transcribed with SMART technology, and amplified by 18 cycles of PCR. Amplified cDNA was purified with AMPure XP beads and quality-assessed on an Agilent Bioanalyzer. Sequencing libraries were prepared using the Nextera XT DNA Library Preparation Kit (Illumina) with a target fragment size of 150–300 bp. Libraries were sequenced on an Illumina NovaSeq 6000 platform with 150-bp paired-end reads to a depth of ≥10 million reads per sample.

Initially, 14 samples were collected across four groups: GV-stage control (n = 4), GV-stage PCOS (n = 3), MII-stage control (n = 3), and MII-stage PCOS (n = 4). Prior to differential expression analysis, principal component analysis (PCA) was performed on log_2_-transformed DESeq2-normalized counts across all 14 samples. One GV-stage control sample (GV_Ctrl_4) was identified as a clear outlier on the PC1–PC2 plot (deviating >3 standard deviations from the group centroid in the full 4-group PCA space) and was excluded to ensure analytical rigor. This sample exhibited markedly elevated expression of ribosomal and metabolic housekeeping genes relative to the other GV control samples, suggesting a possible technical artifact during oocyte collection or library preparation. To confirm that this exclusion did not alter the primary conclusions, a sensitivity analysis retaining GV_Ctrl_4 was performed: the GV-stage differential expression analysis yielded 33 DEGs (versus 35 in the primary analysis), with qualitatively identical conclusions and no change in the key hub genes (*Lsm14b, Sdhb, Cox6a1, Figla, Zp3*), none of which were differentially expressed at the GV stage in either analysis (Supplementary Figure S1). The final dataset comprised 13 samples: GV-stage control (n = 3), GV-stage PCOS (n = 3), MII-stage control (n = 3), and MII-stage PCOS (n = 4).

### 2.7 Read alignment and quantification

Raw sequencing reads were assessed for quality using FastQC (v0.11.9), and adapter sequences were trimmed with Trimmomatic (v0.39; parameters: LEADING:3 TRAILING:3 SLIDINGWINDOW:4:15 MINLEN:36). Trimmed reads were aligned to the mouse reference genome (GRCm38/mm10) using HISAT2 (v2.0.4) (Kim et al., 2019) with default parameters and splice-site annotations derived from Ensembl release 102. Transcript-level abundances were quantified using StringTie (v1.3.4d) (Pertea et al., 2015), generating FPKM values for visualization and raw gene-level read counts for differential expression analysis.

### 2.8 Differential expression analysis

Gene-level count matrices were imported into R (v4.3), and differential expression analysis was performed using the DESeq2 package (v1.40) (Love et al., 2014). Of the 57,132 annotated genes in the reference genome, 40,948 were detected (>0 counts) in at least one sample. After quality filtering (mean normalized count ≥ 10 across all samples), 19,263 genes were retained for differential expression analysis. Four pairwise comparisons were conducted: (1) GV PCOS vs. GV control; (2) MII PCOS vs. MII control; (3) MII control vs. GV control (physiological maturation); and (4) MII PCOS vs. GV PCOS (pathological maturation). Differentially expressed genes (DEGs) were defined by |log_2_ fold change| > 1 and a Benjamini–Hochberg-adjusted p-value (FDR) < 0.05. Genes with mean normalized counts < 10 across all samples were excluded prior to testing. Variance-stabilizing transformation (VST) was applied to normalized counts for PCA and heatmap visualizations.

### 2.9 Maturation trajectory analysis

To characterize stage-specific transcriptional dynamics, a delta–delta log_2_FC framework was applied. For each gene, the maturation-associated log_2_FC was computed in both the control (Δ_normal = log_2_FC_MII_Ctrl/GV_Ctrl) and PCOS (Δ_PCOS = log_2_FC_MII_PCOS/GV_PCOS) conditions. Genes were classified into four trajectory categories: (i) Shared maturation—significant change in both conditions in the same direction; (ii) Impaired in PCOS—significant change in normal maturation but not in PCOS (*p* adj_normal < 0.05, *p* adj_PCOS ≥ 0.05); (iii) Aberrant in PCOS—significant change exclusively in PCOS maturation; and (iv) Reversed in PCOS—significant change in both conditions but in opposite directions. Throughout this manuscript, ‘maternal mRNA clearance’ refers to the programmed degradation of maternally stored transcripts during the GV-to-MII transition—transcripts deposited and stored in the oocyte during oogenesis, regardless of their tissue-specificity. This includes both oocyte-specific genes and ubiquitously expressed metabolic genes (e.g., *Sdhb, Cox6a1*) whose mRNA pools are maternally stored and subject to programmed clearance during meiotic maturation.

### 2.10 Gene ontology and pathway enrichment analysis

Over-representation analysis (GO:BP) was performed using the enrichGO function in the clusterProfiler package (v4.8) (Wu et al., 2021)with the mouse annotation database (org.Mm.eg.db). Significant terms were defined by an FDR < 0.05 with a minimum gene count of 5. Gene Set Enrichment Analysis (GSEA) was performed (Subramanian et al., 2005) using pre-ranked gene lists (ranked by sign (log_2_FC) × −log_10_ (p-value)) against the MSigDB Hallmark gene set collection (v7.5) (Liberzon et al., 2015) and GO:BP gene sets, with 1,000 permutations and a minimum gene set size of 15. Normalized enrichment scores (NES) and Benjamini–Hochberg-adjusted *p*-values are reported.

### 2.11 Weighted gene co-expression network analysis (WGCNA)

**FIGURE 1:**
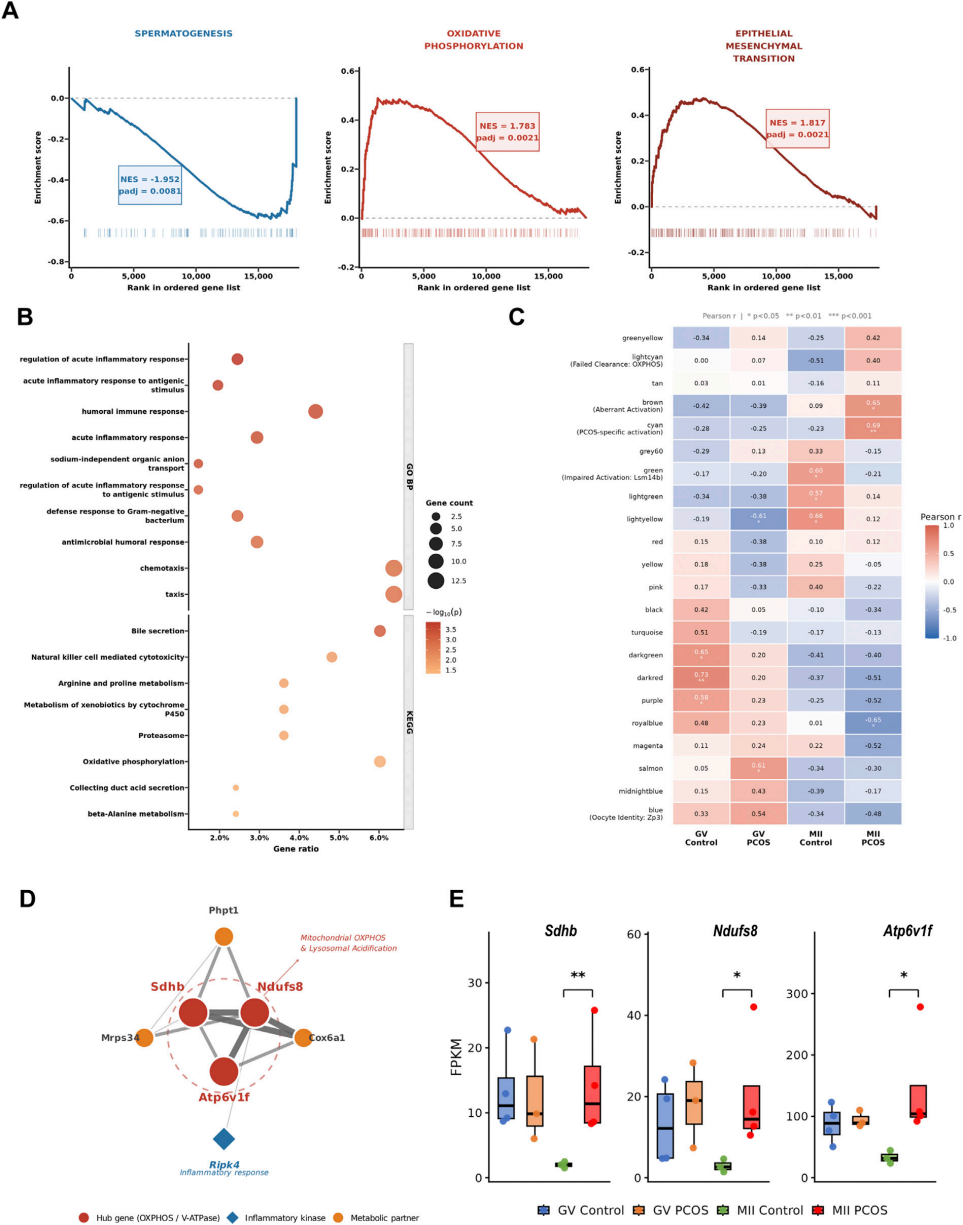
Pathway enrichment and co-expression network analyses reveal coordinated mitochondrial dysregulation and module-level transcriptomic reorganization in PCOS MII oocytes. **(A)** Gene Set Enrichment Analysis (GSEA) evaluating global transcriptomic shifts in MII oocytes from the PCOS group. Significant enrichments are shown for selected Hallmark gene sets. Spermatogenesis is negatively enriched (NES = −1.952, *p*adj = 0.008), reflecting the depletion of shared germ-cell transcriptional programs in PCOS MII oocytes; the MSigDB Hallmark Spermatogenesis gene set contains conserved germ-cell regulatory genes active in both male and female germlines, and its negative enrichment is consistent with the collapse of oocyte identity gene networks. Oxidative Phosphorylation (NES = +1.783, *p*adj = 0.002) and Epithelial-Mesenchymal Transition (NES = +1.817, *p*adj = 0.002) are positively enriched in PCOS MII oocytes, indicating aberrant retention or upregulation of these gene programs. NES, normalized enrichment score; *p*adj, adjusted p-value. **(B)** Bubble plot of Gene Ontology (GO) biological processes and KEGG pathways enriched among the DEGs. Node size represents the gene count, and color intensity corresponds to statistical significance. **(C)** Heatmap of module–trait Pearson correlations generated by WGCNA (22 modules × 4 sample-group traits). Modules are annotated with their dominant biological function, inferred from GO-BP enrichment of module member genes and representative hub gene identity. Four modules show biologically interpretable stage-specific patterns: the lightcyan module (Failed Clearance: OXPHOS) shows a negative correlation with MII Control (r = −0.51) and a positive trend with MII PCOS (r = +0.40), consistent with impaired clearance of mitochondrial transcripts during maturation; the green module (Impaired Activation: *Lsm14b*) is positively correlated with MII Control (r = +0.60, *p* < 0.05), reflecting its physiological activation during normal maturation that is absent in PCOS; the cyan and brown modules show the strongest positive correlations with MII PCOS (r = +0.69, *p* < 0.01 and r = +0.65, *p* < 0.05, respectively), representing aberrant transcriptional programs specific to PCOS maturation. Asterisks denote statistical significance: *p < 0.05, **p < 0.01, ***p < 0.001 (Pearson correlation, across n = 13 samples). **(D)** Protein-Protein Interaction (PPI) network focusing on core dysregulated genes. Key hub genes center on mitochondrial OXPHOS and lysosomal acidification (e.g., *Ndufs8, Sdhb, Atp6v1f*, colored in red) alongside inflammatory signaling (*Ripk4*). Node size reflects degree of connectivity; edge width is proportional to STRING interaction confidence score. **(E)** Boxplots depicting the expression dynamics (FPKM values) of representative hub genes (*Sdhb, Ndufs8, Atp6v1f*) across maturation stages, demonstrating specific dysregulation upon reaching the MII stage. *p < 0.05; **p < 0.01.

Co-expression network analysis was performed using the WGCNA package (v1.72) (Langfelder and Horvath, 2008). Variance-stabilizing transformation (VST)-normalized expression values from all 13 samples were used as input. The 5,000 most variably expressed genes (by median absolute deviation) were selected for network construction. A soft-thresholding power of β = 8 was selected based on the scale-free topology criterion (R^2^ = 0.879). Signed hybrid co-expression modules were identified using the blockwiseModules function (networkType = ‘signed hybrid’, TOMType = ‘signed’, minModuleSize = 30, mergeCutHeight = 0.25, deepSplit = 2), yielding 22 co-expression modules. Module eigengene–trait correlation analysis was performed using Pearson correlation between module eigengenes and sample-group trait vectors (GV Control, GV PCOS, MII Control, MII PCOS), with statistical significance assessed by two-tailed t-test. Module biological annotations were assigned based on the dominant GO Biological Process enrichment term (clusterProfiler enrichGO, FDR < 0.05) of module member genes; for modules lacking significant GO enrichment, annotations were based on the identity of the highest-kME hub gene. Hub genes were defined as module members with module membership (kME) > 0.80. Among the four biologically interpretable modules highlighted in Figure 1C, the lightcyan (‘Failed Clearance: OXPHOS’) and green (‘Impaired Activation: Lsm14b’) modules were annotated based on their dominant GO-BP enrichment terms (clusterProfiler enrichGO, FDR < 0.05); the cyan and brown modules were annotated based on their strong positive correlations with the MII PCOS group (r = +0.69, *p* < 0.01 and r = +0.65, *p* < 0.05, respectively), representing aberrant PCOS-specific activation programs.

### 2.12 Low-input RNA extraction and reverse transcription

Given the scarce nature of the material, total RNA was extracted from pools of 10 oocytes per sample using the Super FastPure Cell RNA Isolation Reagent (Vazyme Biotech, RC102, China). To maximize cDNA yield, the entire eluted RNA volume was immediately subjected to reverse transcription using the HiScript III RT SuperMix for qPCR (Vazyme Biotech, R323, China) in a 20 μL reaction volume (37 °C for 15 min, 85 °C for 5 s). The resulting cDNA product was then diluted tenfold and used for PCR amplification.

### 2.13 Quantitative real-time PCR (qRT-PCR)

Quantitative real-time PCR (qRT-PCR) was carried out on an Applied Biosystems (ABI) 7500 real-time PCR system in a 20 μL reaction system containing 10 μL ChamQ Universal SYBR qPCR Master Mix (Vazyme Biotech, Q711, China), 0.4 μL each of forward and reverse primers (10 μM), and 2 μL cDNA template, under the following cycling conditions: initial denaturation at 95 °C for 5 min, followed by 40 cycles of denaturation at 95 °C for 15 s and annealing/extension at 60 °C for 34 s. Each sample was run in triplicate, and the entire experiment was repeated three times. The cycle threshold (CT) values were recorded for all groups. Gapdh was used as the internal reference gene. The suitability of Gapdh as a reference gene was confirmed by RNA-seq data, which showed no significant differential expression of Gapdh between PCOS and control groups at either the GV stage (log_2_FC = 0.003, *p*adj = 1.0) or the MII stage (log_2_FC = 0.010, *p*adj = 0.993). The observed variation in Gapdh normalized counts across all samples reflects the expected stage-dependent transcriptomic remodeling between GV and MII oocytes, which is fully accounted for by the ΔΔCt normalization framework using the GV Control group as calibrator. Relative mRNA expression levels were calculated using the 2^^-ΔΔCt^ method (Livak and Schmittgen, 2001), with the GV-stage Control group serving as the calibrator (RQ = 1). The primers used are listed in Table 1.

**TABLE 1:** RT-qPCR primer sequences.

Gene	Forward (5'→3′)	Reverse (5'→3′)	Product	Accession
*Sdhb*	TCC​AGA​GAC​GAC​TTC​ACA​GA	TGT​GCA​GTT​CAT​GAT​GGT​GT	87 bp	NM_023374
*Cox6a1*	ATG​CTC​AAC​GTG​TTC​CTC​AA	AGG​GTT​GTG​GAA​GAG​GGT​AT	132 bp	NM_007748
*Lsm14b*	ATT​GTT​GGA​CAG​TCT​GGC​TC	ACC​ACA​TTT​GCT​GGG​ATG​AA	183 bp	NM_026304
*Figla*	AGA​AGC​GAA​GGT​CTC​AGA​GA	GAG​CTC​TCT​AGT​CGA​GGA​CA	91 bp	NM_012013
*Gapdh*	TCA​AGA​AGG​TGG​TGA​AGC​AG	GAA​GAG​TGG​GAG​TTG​CTG​TT	106 bp	NM_008084

### 2.14 Western blot analysis

To validate key transcriptomic findings at the protein level, Western blot analysis was performed on Lsm14b, Sdhb, and Figla proteins in GV and MII oocytes from control and PCOS mice. For each lane, lysate from 150 oocytes was prepared by pooling oocytes from three independent experiments. Oocytes were lysed in RIPA buffer (50 mM Tris-HCl pH 7.4, 150 mM NaCl, 1% NP-40, 0.5% sodium deoxycholate, 0.1% SDS) supplemented with protease inhibitor cocktail (Roche) on ice for 30 min. Protein concentration was determined using the BCA Protein Assay Kit (Thermo Fisher Scientific). Equal amounts of protein were separated by 4%–20% SDS-PAGE and transferred to PVDF membranes (Millipore). Membranes were blocked with 5% non-fat milk in TBST for 1 h at room temperature, then incubated overnight at 4 °C with primary antibodies against Lsm14b (1:1,000; Abcam), Sdhb (1:1,000; Abcam), Figla (1:500; Abcam), and Gapdh (1:5,000; Proteintech). After washing, membranes were incubated with HRP-conjugated secondary antibodies for 1 h at room temperature. Bands were visualized using enhanced chemiluminescence (ECL) reagent and quantified using ImageJ software. Band intensities were normalized to Gapdh loading control. Data are presented as mean ± SEM from n = 3 biological replicates. Statistical significance was determined using a two-tailed Student’s t-test.

### 2.15 Statistical analysis

Statistical analyses were performed in R (v4.3). For RT-qPCR data, statistical significance between conditions at each maturation stage was determined using a two-tailed Welch’s t-test. Quantitative data are presented as mean ± standard deviation (SD). For transcriptomic comparisons involving non-normally distributed gene expression distributions, Wilcoxon rank-sum tests were applied. A *p*-value < 0.05 was considered statistically significant. Figures were generated using ggplot2 (v3.4), ComplexHeatmap (v2.14), and ggprism (v1.0.5).

## 3 Results

### 3.1 Establishment and validation of the DHEA-induced PCOS mouse model

**FIGURE 2:**
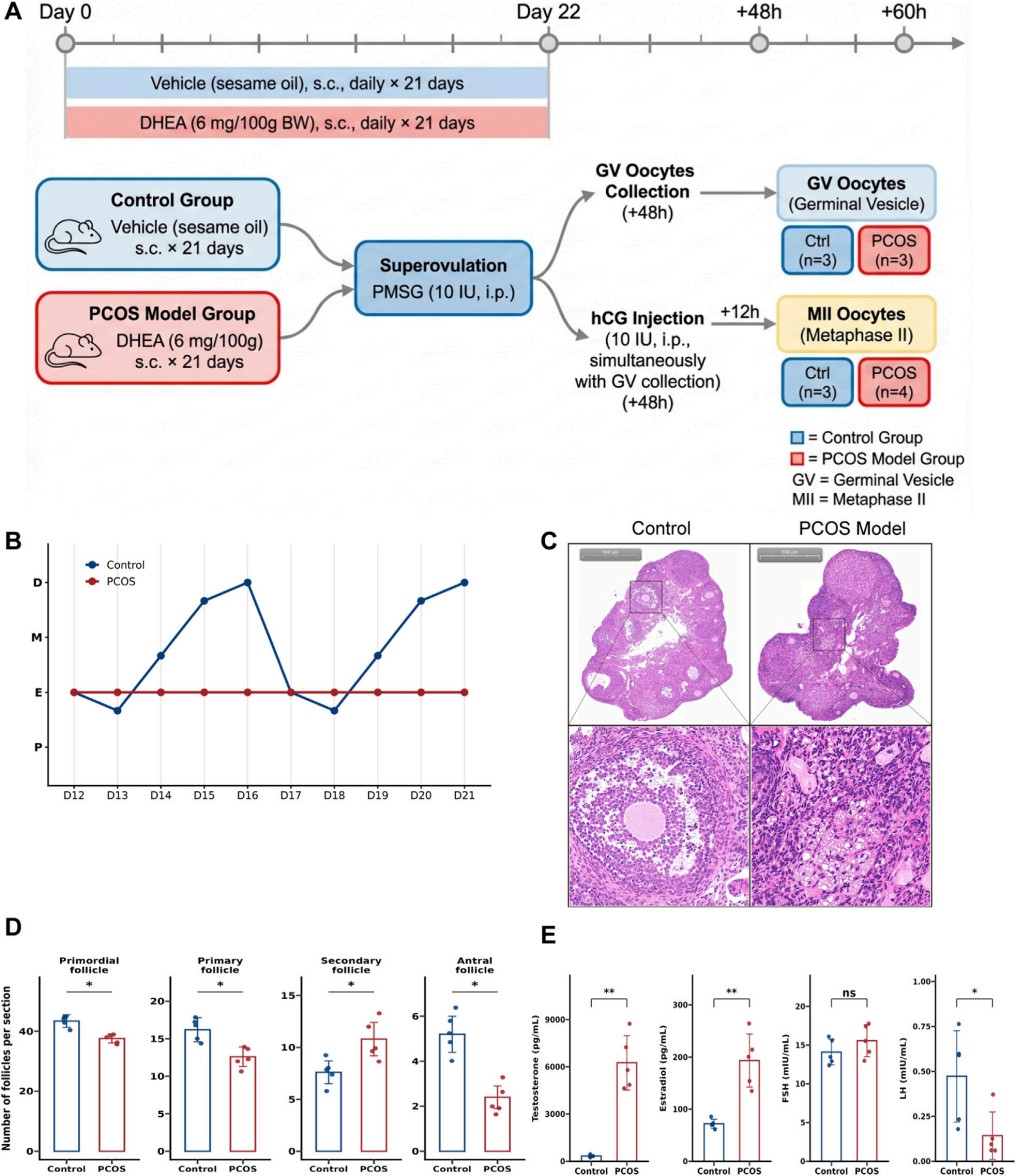
Phenotypic, hormonal, and histological characterization of the DHEA-induced PCOS mouse model. **(A)** Schematic diagram of the experimental design. Mice were subcutaneously injected with DHEA or vehicle for 21 days to establish the PCOS model, followed by superovulation for oocyte collection. **(B)** Representative estrous cycle patterns of the Control and PCOS model groups during days 12–21 of DHEA administration, monitored by vaginal smears (P: Proestrus; E: Estrus; M: Metestrus; D: Diestrus). **(C)** Representative images of hematoxylin and eosin (H&E)-stained ovarian cross-sections from Control and PCOS mice. PCOS ovaries display characteristic histological features including an increased proportion of cystic follicles and an absence of corpora lutea. Scale bar, 500 μm. **(D)** Quantitative analysis of the number of primordial, primary, secondary, and antral follicles per ovarian section. Data are presented as mean ±SD. *p < 0.05, Mann-Whitney U test. **(E)** Measurement of serum hormone levels, including testosterone, estradiol, follicle-stimulating hormone (FSH), and luteinizing hormone (LH). Data are presented as mean ±SD (n = 5 mice per group). Statistical significance was determined using the Mann-Whitney U test (*p < 0.05, **p < 0.01; ns, not significant).

To investigate the molecular alterations in oocytes under PCOS conditions, we established a well-characterized PCOS mouse model via continuous subcutaneous injection of DHEA for 21 days. GV and MII oocytes were subsequently collected following superovulation (Figure 2A). The successful establishment of the model was comprehensively validated through estrous cycle monitoring, ovarian histopathology, and serum hormone profiling.

As shown in Figure 2B, control mice maintained regular estrous cycles, whereas DHEA-treated mice completely lost estrous cyclicity, indicating ovulatory dysfunction. Histological examination via H&E staining revealed characteristic morphological abnormalities in PCOS ovaries, notably the accumulation of cystic-like follicles and an apparent lack of corpora lutea (Figure 2C). Serum endocrine profiling revealed the hormonal hallmarks of PCOS: testosterone and estradiol (E2) levels were significantly elevated in the PCOS group compared to controls (*p* < 0.01), while follicle-stimulating hormone (FSH) levels remained unaltered and luteinizing hormone (LH) levels were significantly decreased (Figure 2D). The observed LH suppression is consistent with androgen-mediated negative feedback on the hypothalamic-pituitary-gonadal (HPG) axis, a hallmark of exogenous androgen-induced PCOS models. It should be noted that this LH suppression contrasts with the elevated LH typically observed in clinical PCOS, which reflects the distinct pathophysiology of endogenous hyperandrogenism driven by altered GnRH pulsatility, rather than exogenous androgen administration. Quantitative analysis of follicular dynamics further confirmed significant alterations in follicle composition, including a significant decrease in the number of primordial, primary, and antral follicles, accompanied by an increase in secondary follicles in the PCOS group (*p* < 0.05, Figure 2E), indicating severe follicular arrest. Together, these multi-dimensional phenotypic, histological, and endocrine alterations collectively establish the fidelity of the DHEA-induced PCOS model and provide a validated platform for subsequent transcriptomic investigation.

### 3.2 PCOS-induced transcriptomic dysregulation erupts specifically during the GV-to-MII meiotic transition

**FIGURE 3:**
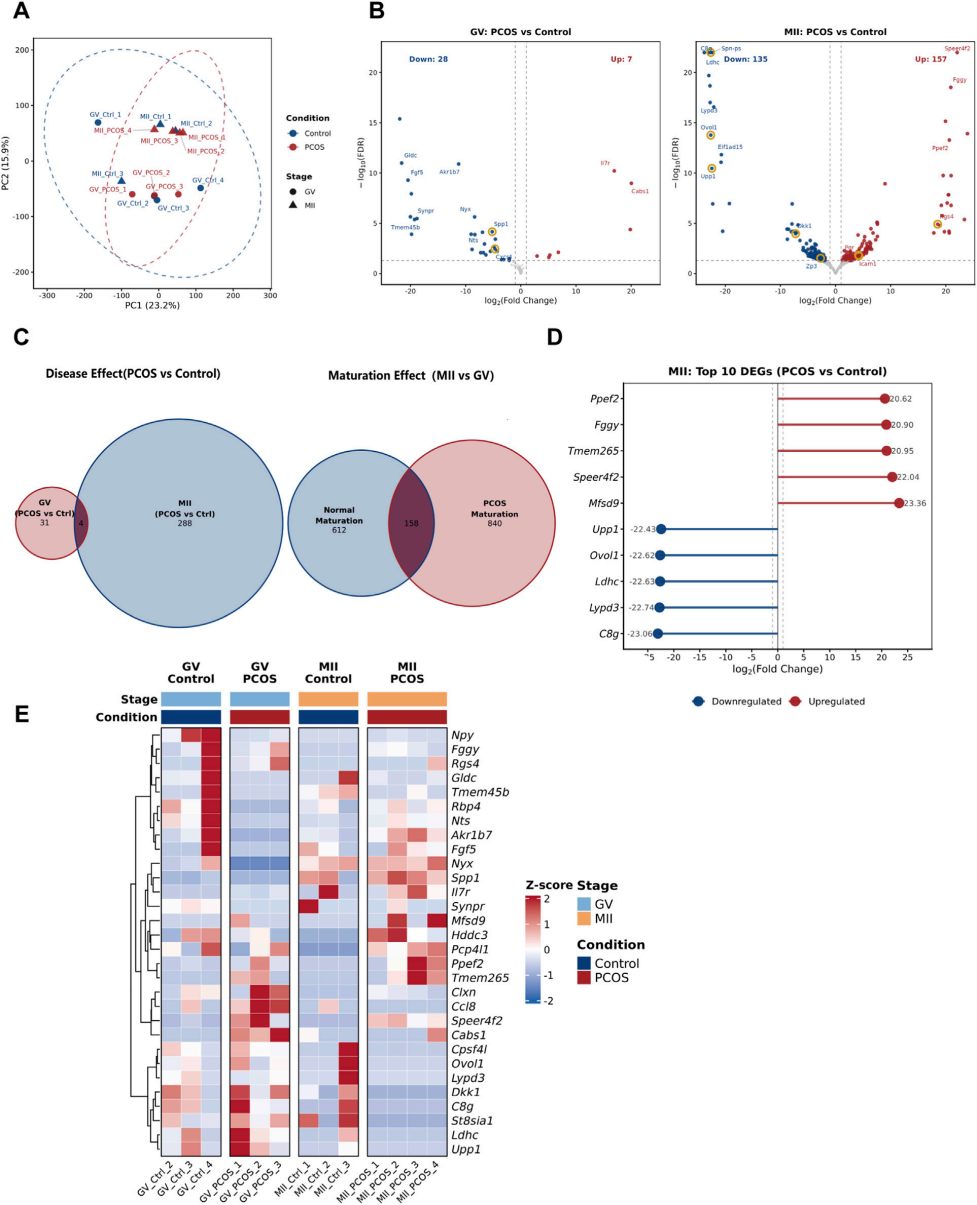
Transcriptomic profiling reveals that PCOS-induced gene expression dysregulation erupts specifically during the GV-to-MII transition. **(A)** Principal component analysis (PCA) plot of RNA-seq data from GV and MII oocytes. PC1 (23.6% variance) primarily reflects developmental stage, while PC2 (16.4% variance) captures disease-associated variation. **(B)** Volcano plots illustrating differentially expressed genes (DEGs) in PCOS vs. Control groups at the GV stage (left) and MII stage (right). Thresholds: |log_2_FC| > 1 and FDR < 0.05. Representative key genes are highlighted. **(C)** Venn diagrams illustrating the stage-specificity of PCOS-induced transcriptomic dysregulation. Left: The minimal overlap of disease-induced DEGs between GV and MII stages highlights the stage-specific nature of PCOS-induced transcriptomic dysregulation, with the majority of DEGs unique to the MII stage. Right: Overlap of maturation-associated dynamically expressed genes (MII vs. GV) in Normal and PCOS conditions. **(D)** Lollipop plot highlighting the top DEGs with the most extreme log2 fold changes in MII oocytes from the PCOS group. **(E)** Heatmap displaying the relative expression levels (Z-score) of representative core DEGs across all four experimental groups, confirming that major transcriptional divergence is predominantly confined to the MII stage.

To map the molecular landscape of oocyte decline in PCOS, we performed RNA-sequencing (RNA-seq) on GV and MII oocytes. Principal component analysis (PCA) revealed that transcriptomic profiles were primarily driven by the developmental stage along PC1 (23.6%), while the disease condition induced separation along PC2 (16.4%). Notably, the transcriptomic distance between Control and PCOS samples was minimal at the GV stage but expanded markedly at the MII stage (Figure 3A), suggesting a stage-specific amplification of pathological effects.

Differential expression analysis confirmed this developmental window vulnerability. In GV oocytes, the PCOS condition induced minimal alterations, yielding only 35 DEGs. However, in MII oocytes, the transcriptomic divergence became pronounced, with the number of DEGs expanding to 292 (157 upregulated and 135 downregulated; Figure 3B). Venn diagram analysis highlighted that this disruption was highly stage-specific, with minimal overlap of DEGs between the GV and MII stages (Figure 3C, left). Furthermore, comparison of maturation-associated dynamic genes revealed that normal oocytes undergo coordinated transcriptomic remodeling involving 2,152 dynamically expressed genes during the GV-to-MII transition, whereas PCOS oocytes show a substantially altered dynamic program with only 311 genes shared between the two conditions (Figure 3C, right), underscoring the pervasive disruption of physiological maturation programs.

To pinpoint the most severely affected targets, we ranked the MII-specific DEGs by their absolute fold changes (Figure 3D). Unsupervised hierarchical clustering of these top dysregulated transcripts and representative core genes confirmed that the major transcriptional divergence strictly occurred in MII-PCOS oocytes (Figure 3E). These data demonstrate that the pathological impact of PCOS on the oocyte transcriptome does not originate as a baseline defect at the GV stage, but rather erupts acutely and specifically during the process of meiotic maturation, defining a critical developmental window of vulnerability.

### 3.3 Network analysis uncovers mitochondrial and metabolic dysregulation as key drivers of MII oocyte decline

To elucidate the functional consequences of these MII-specific transcriptomic disruptions, we performed Gene Set Enrichment Analysis (GSEA). The analysis revealed significant enrichment in Hallmark gene sets, including negative enrichment of Spermatogenesis (NES = −1.952, *p*adj = 0.008), and positive enrichment of Oxidative Phosphorylation (OXPHOS; NES = +1.783, *p*adj = 0.002) and Epithelial-Mesenchymal Transition (EMT; NES = +1.817, *p*adj = 0.002) (Figure 1A). The negative enrichment of the Spermatogenesis gene set reflects the depletion of shared germ-cell transcriptional programs in PCOS MII oocytes: the MSigDB Hallmark Spermatogenesis collection contains genes representing conserved germ-cell regulatory programs active in both male and female germlines, and its negative enrichment is consistent with the broader collapse of oocyte identity gene networks identified by trajectory analysis. The positive enrichment of the EMT gene set likely reflects aberrant activation of cytoskeletal remodeling and cell polarity programs during PCOS maturation, consistent with prior reports of cytoskeletal dysregulation in PCOS oocytes. Gene Ontology (GO) and KEGG functional categorizations further clustered the DEGs into pathways associated with inflammatory responses, chemotaxis, and proteasome functions (Figure 1B).

To move beyond single-gene analyses and identify core co-regulated functional clusters, we performed Weighted Gene Co-expression Network Analysis (WGCNA), yielding 22 co-expression modules. Module–trait correlation analysis revealed a coordinated, multi-module dysregulation pattern that recapitulates the dual-layered transcriptomic failure identified by trajectory analysis (Figure 1C). The lightcyan module, annotated as ‘Failed Clearance: OXPHOS’ based on GO-BP enrichment of its member genes, showed a negative correlation with MII Control (r = −0.51) and a positive trend with MII PCOS (r = +0.40), consistent with the pathological retention of mitochondrial transcripts during PCOS maturation. The green module, whose hub gene is *Lsm14b*, was positively correlated with MII Control (r = +0.60, *p* < 0.05) but not with MII PCOS (r = −0.21), reflecting the physiological activation of the mRNA decay machinery that is selectively abrogated in PCOS. The cyan and brown modules showed the strongest positive correlations with MII PCOS (r = +0.69, *p* < 0.01 and r = +0.65, *p* < 0.05, respectively), representing aberrant transcriptional programs that are specifically activated during PCOS maturation. Notably, while disease-induced DEGs at the GV stage were minimal (n = 35), two modules (lightyellow and salmon) exhibited significant correlations with the GV PCOS group (r = −0.61 and r = +0.61, respectively; *p* < 0.05), suggesting that subtle, coordinated transcriptional perturbations may already exist at the GV stage at the network level, although these do not reach the threshold of robust single-gene differential expression. The dramatic MII-stage divergence likely reflects the unmasking and amplification of this latent network instability during the post-transcriptional remodeling demanded by meiotic maturation.

To identify structural hub regulators among these MII-stage transcriptomic disruptions, we submitted the 292 significantly dysregulated MII-stage genes to the STRING database for protein–protein interaction (PPI) network construction. As illustrated in Figure 1D, the resulting network architecture was highly enriched for mitochondrial OXPHOS and lysosomal acidification components, prominently featuring *Sdhb, Ndufs8,* and *Atp6v1f* as the highest-connectivity hub genes, alongside the inflammatory kinase *Ripk4* which bridges the metabolic cluster to immune signaling. Notably, these OXPHOS hub genes are also co-classified within the lightcyan (‘Failed Clearance: OXPHOS’) module identified by WGCNA, providing convergent evidence from two independent analytical frameworks. We then tracked the absolute expression trajectories of these hub genes across the maturation window (Figure 1E). Their expression levels were comparable between control and PCOS oocytes at the GV stage (all *p* > 0.05, Wilcoxon rank-sum test), but diverged significantly upon reaching the MII stage (*p* < 0.05 to *p* < 0.01). Notably, while these OXPHOS/V-ATPase hub transcripts were physiologically downregulated in control MII oocytes—consistent with their classification as maternal transcripts subject to programmed clearance during meiotic maturation—they remained aberrantly elevated in PCOS MII oocytes, indicating a failure of maternal mRNA clearance rather than a primary transcriptional upregulation. This structural mapping identifies mitochondrial OXPHOS and lysosomal acidification hubs as central nodes of the MII-PCOS transcriptomic collapse.

### 3.4 Dynamic trajectory analysis reveals stalled clearance programs and aberrant transcript dynamics

**FIGURE 4:**
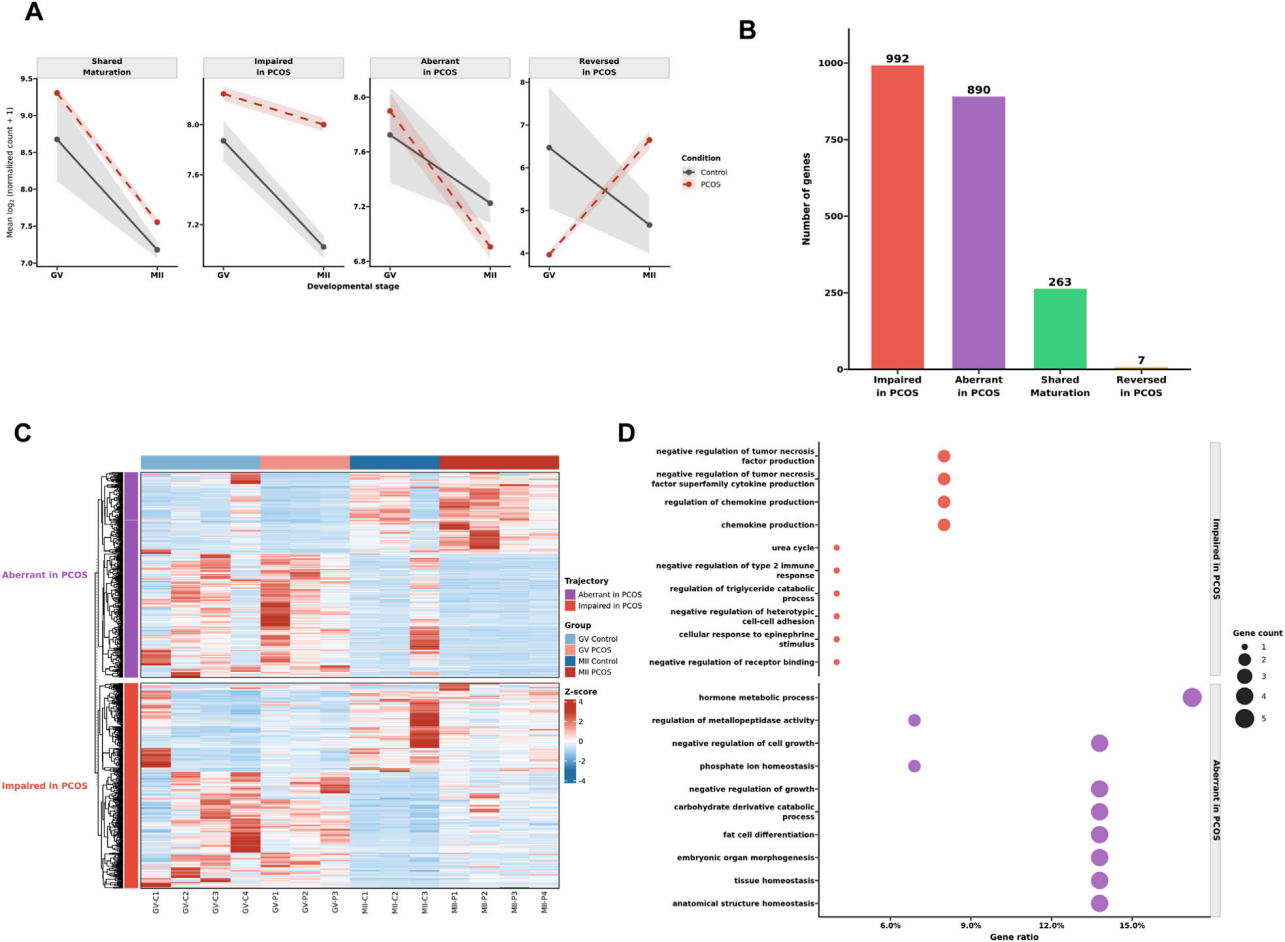
Dynamic trajectory analysis reveals disrupted maturation programs and aberrant transcript dynamics in PCOS oocytes. **(A)** Line plots categorizing all dynamically expressed genes into four distinct trajectories across GV to MII transition: Shared Maturation, Impaired in PCOS, Aberrant in PCOS, and Reversed in PCOS. Solid grey lines denote physiological maturation; dashed red lines represent altered dynamics in PCOS. **(B)** Bar chart quantifying the gene distribution among the four trajectories. The majority of dynamically regulated genes fall within the ‘Impaired’ or ‘Aberrant’ categories. **(C)** Heatmap with hierarchical clustering validating the expression profiles of the two dominant pathological gene sets (“Aberrant in PCOS”, purple; “Impaired in PCOS”, red) across individual biological replicates. Expression values are Z-score scaled. **(D)** Split bubble plot detailing the distinct Gene Ontology (GO) Biological Process enrichments for the derailed trajectories. The “Impaired” trajectory comprises transcripts that undergo normal downregulation during maturation in controls but fail to be cleared in PCOS oocytes; these are enriched for OXPHOS components and mitochondrial metabolic processes. The “Aberrant” trajectory comprises transcripts exhibiting aberrant upregulation or retention in PCOS MII oocytes, enriched for oogenesis-related processes.

Having established that PCOS pathology erupts during the MII stage, we systematically mapped the perturbation of physiological gene expression dynamics during the GV-to-MII transition. We categorized all dynamically expressed transcripts (n = 2,152) into four longitudinal trajectories: “Shared Maturation” (n = 263), “Impaired in PCOS” (n = 992), “Aberrant in PCOS” (n = 890), and “Reversed in PCOS” (n = 7) (Figure 4A).

Quantitative distribution revealed that the vast majority of dynamic genes (87.5%) were diverted into the two dominant pathological trajectories (Figure 4B). To validate these patterns, a hierarchical clustering heatmap confirmed that transcriptomic divergence erupted strictly upon reaching the MII stage (Figure 4C).

Importantly, split GO enrichment analysis decoded the distinct biological natures of these derailments (Figure 4D). The “Impaired” trajectory (n = 992) comprised two functionally related subsets: transcripts that undergo physiological downregulation during normal maturation but fail to be cleared in PCOS oocytes (n = 558), and transcripts that normally undergo upregulation but fail to be activated in PCOS (n = 434). Together, these retained and unactivated transcripts were heavily enriched for OXPHOS components and mitochondrial metabolic processes. The “Aberrant” trajectory (n = 890) comprised transcripts that exhibit abnormal expression dynamics specifically in PCOS MII oocytes, including genes that are prematurely depleted to a far greater extent than the physiological decline observed in controls (e.g., oocyte identity genes *Figla* and *Zp3*), as well as genes that are inappropriately upregulated. These were functionally enriched for oogenesis and female gamete generation processes. These findings reveal that PCOS simultaneously and paradoxically disrupts two opposing but complementary aspects of the maternal transcriptome remodeling program: the timely clearance and activation of metabolic transcripts, and the preservation of oocyte identity gene networks—a dual failure that collectively undermines the transcriptomic competence required for successful meiotic maturation.

### 3.5 Global failure of maternal mRNA clearance and impaired activation of decay machinery

**FIGURE 5:**
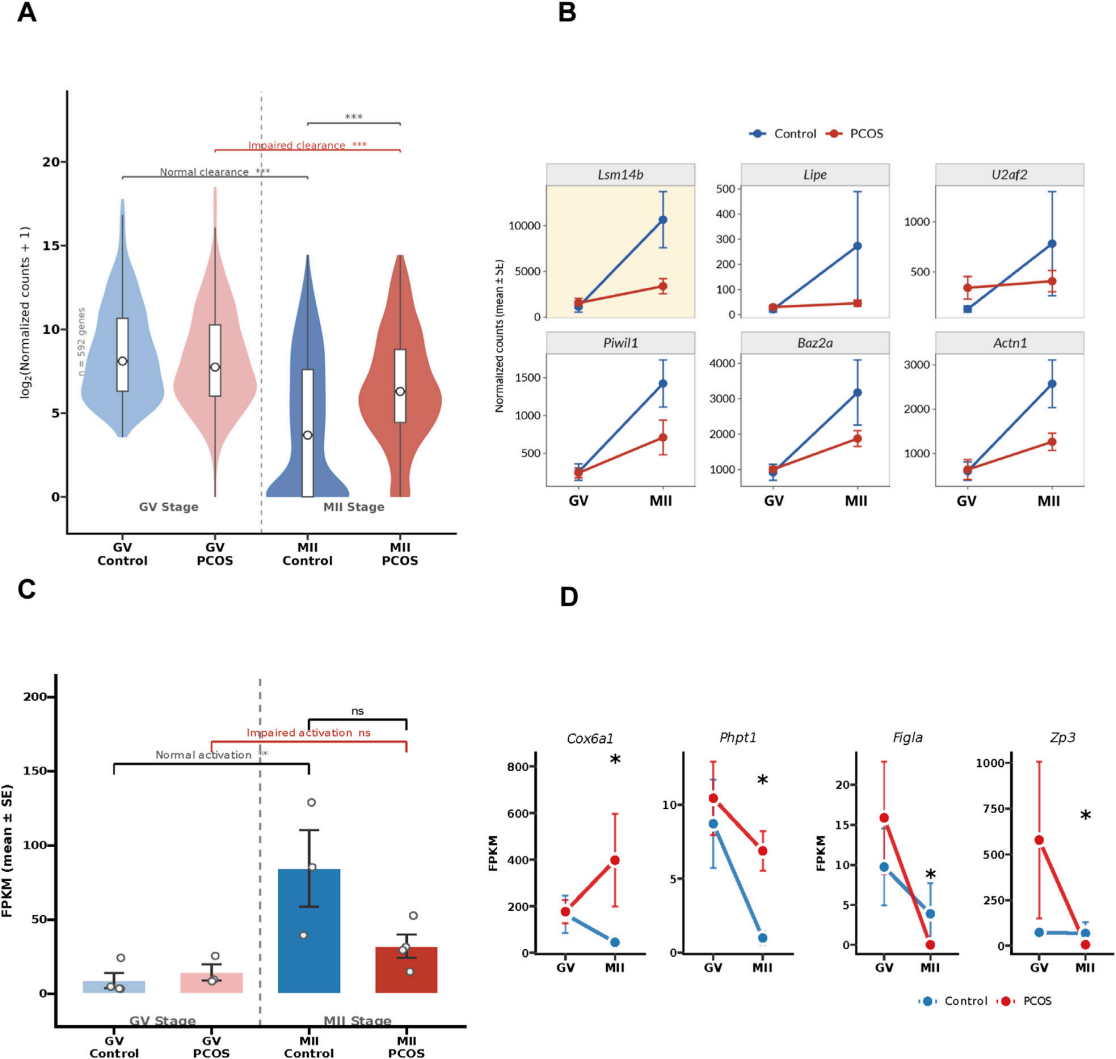
Impaired maternal mRNA clearance and defective activation of key regulatory factors define the transcriptomic collapse in PCOS MII oocytes. **(A)** Violin plots quantifying maternal mRNA clearance efficiency. The violin plot displays all 874 physiological decay targets (defined as transcripts with *p*adj < 0.05 and log2FC < −1 during normal GV-to-MII maturation). Of these 874 genes, 592 showed defective clearance in PCOS oocytes (440 fully impaired + 152 partially impaired), while 282 were properly cleared. Statistical significance was determined by Wilcoxon rank-sum test (***p < 0.001). **(B)** Dynamic expression trajectories of critical maturation-associated regulatory factors exhibiting the “impaired activation” pattern in PCOS oocytes, including representative mRNA-regulatory factors (*Lsm14b, U2af2, Piwil1, Baz2a*) and co-regulated transcripts (*Lipe, Actn1*). **(C)** Detailed expression profile (FPKM) of *Lsm14b*, a central P-body assembly factor, highlighting its significant failure to activate in MII PCOS oocytes (**p < 0.01, Control MII vs. PCOS MII). **(D)** Expression dynamics of specific representative transcripts. Left: “Failed Clearance” of maternal metabolic transcripts (*Cox6a1, Phpt1*), which abnormally accumulate in MII PCOS oocytes. Right: “Premature Depletion” of essential oocyte identity genes (*Figla, Zp3*), illustrating two distinct modes of transcript dysregulation in PCOS MII oocytes. *p < 0.05.

The programmed clearance of maternal mRNAs is a fundamental prerequisite for successful oocyte maturation. Given the extensive transcript retention observed in the trajectory analysis, we quantified global maternal mRNA clearance efficiency across all four experimental groups. A large proportion of physiological decay targets (n = 874 genes; defined as *p* adj < 0.05 and log_2_FC < −1 during normal maturation) exhibited severely impaired degradation in PCOS oocytes during the GV-to-MII transition (*p* < 0.001, Figure 5A), demonstrating a systemic failure of the maternal mRNA clearance program.

To uncover the regulatory root of this clearance failure, we investigated the dynamic expression of maturation-associated regulatory factors. Under normal physiological conditions, key factors associated with mRNA decay and P-body assembly—including *Lsm14b, U2af2, Piwil1*, and *Baz2a*, along with co-regulated factors Lipe and Actn1—undergo robust upregulation during the GV-to-MII transition. However, in PCOS oocytes, these factors exhibited a completely blunted “impaired activation” pattern, failing to upregulate during maturation (Figure 5B). Specifically, *Lsm14b*, a central P-body assembly factor crucial for RNA degradation, failed entirely to execute its normal MII-stage activation in PCOS oocytes (*p* < 0.01, Figure 5C), implicating defective RNA decay machinery as a proximal mechanism underlying the observed clearance failure.

This collapse of the decay machinery directly translated into terminal transcriptomic consequences at the level of individual transcripts (Figure 5D). We observed a dual-layered disruption: on one hand, the pathological “Failed Clearance” of maternal metabolic transcripts (e.g., *Cox6a1, Phpt1*), which abnormally accumulated in MII PCOS oocytes (*p* adj < 0.05); on the other hand, the “Premature Depletion” of essential oocyte identity genes (e.g., *Figla, Zp3*), which were dramatically reduced in PCOS MII oocytes (*p* adj < 0.05). Notably, while *Figla* undergoes physiological downregulation during normal GV-to-MII maturation, its expression in PCOS MII oocytes was reduced to near-undetectable levels, representing a pathological amplification of the normal decay program. These two opposing dysregulations—aberrant retention of metabolic transcripts and premature loss of oocyte identity genes—collectively demonstrate that PCOS disrupts the coordinated post-transcriptional regulation required for oocyte developmental competence.

### 3.6 RT-qPCR validation confirms stage-specific dysregulation of hub transcripts and supports the working model

**FIGURE 6:**
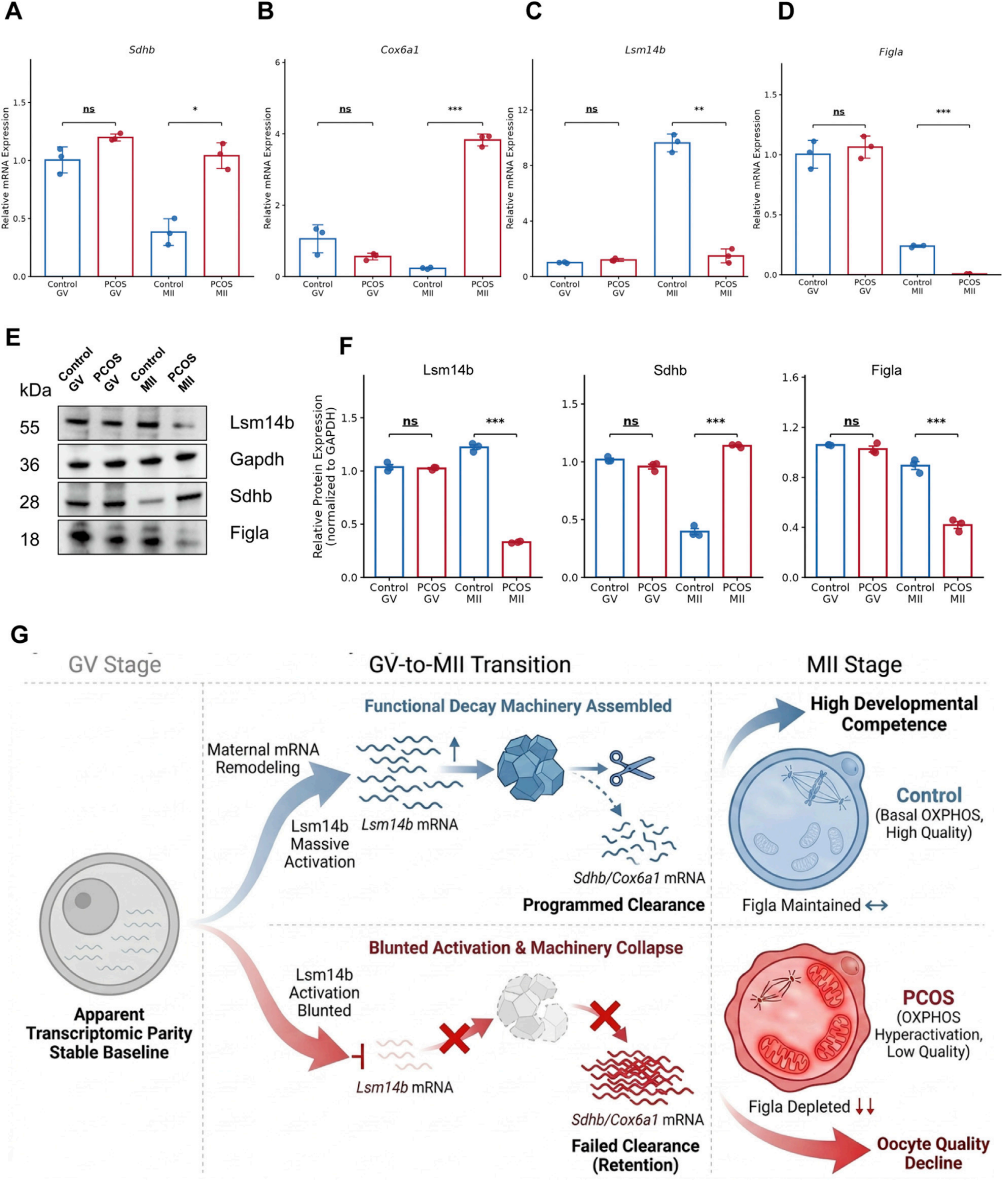
Experimental validation of transcriptomic derailment and the proposed working model of PCOS oocyte maturation failure. **(A–D)** RT-qPCR validation of the relative mRNA expression of metabolic hub genes (*Sdhb, Cox6a1*), the mRNA-decay factor (*Lsm14b*), and the oocyte identity gene (*Figla*) across the GV-to-MII transition. Relative quantification (RQ) was calculated using the 2^(−ΔΔCt) method, with the Control GV group serving as the calibrator (RQ = 1). Data are presented as mean ± SD of three independent biological replicates. Statistical significance between conditions at each stage was determined using a two-tailed Welch’s t-test (*p < 0.05, **p < 0.01, ***p < 0.001; ns, not significant). The data demonstrate apparent transcriptomic parity at the GV stage, followed by severe dysregulation specifically unmasked at the MII stage. **(E)** Representative Western blot images showing protein expression of Lsm14b, Sdhb, and Figla in GV and MII oocytes from control and PCOS mice. Gapdh was used as the loading control. Each lane was loaded with lysate from 150 oocytes pooled from three independent biological replicates. **(F)** Quantification of Western blot band intensities for Lsm14b, Sdhb, and Figla, normalized to Gapdh. Data are presented as mean ± SEM from n = 3 biological replicates. Statistical significance was determined using a two-tailed Student’s t-test. Lsm14b protein was significantly reduced in PCOS MII oocytes compared to controls (3.7-fold, *p* < 0.001; ***), Sdhb protein was aberrantly elevated in PCOS MII oocytes (2.9-fold, *p* < 0.001; ***), and Figla protein was markedly depleted in PCOS MII oocytes (2.1-fold, *p* < 0.001; ***). GV-stage differences: Lsm14b ns (*p* = 0.644), Sdhb ns (*p* = 0.065), Figla ns (*p* = 0.312). **(G)** The proposed working model illustrating the stage-specific transcriptomic collapse in PCOS oocytes. During the critical GV-to-MII transition, the blunted activation of Lsm14b in PCOS oocytes impairs the assembly of the functional decay machinery. This regulatory failure leads to the pathological retention of metabolic transcripts (*Sdhb, Cox6a1*), which occurs concomitantly with the aberrant hyper-depletion of identity genes (*Figla*). Together, these dual transcriptomic disruptions establish a molecular signature indicative of OXPHOS hyperactivation, ultimately contributing to oocyte quality decline.

To independently validate the transcriptomic findings at the single-gene level and confirm the stage-specificity of the identified dysregulation, we performed RT-qPCR on four representative hub transcripts—*Sdhb, Cox6a1, Lsm14b, and Figla*—across GV and MII oocytes from control and PCOS mice (Figures 6A–D).

Consistent with the RNA-seq data, none of the four genes showed significant expression differences between control and PCOS oocytes at the GV stage (all *p* ≥ 0.05), confirming that the transcriptomic landscape is largely intact prior to meiotic maturation. This GV-stage equivalence is critical: it establishes that the downstream MII-stage divergences reflect a failure of the maturation process itself, rather than a pre-existing baseline defect.

Upon meiotic maturation to the MII stage, all four transcripts exhibited significant and biologically coherent dysregulation in PCOS oocytes. The mitochondrial OXPHOS hub gene *Sdhb* showed significantly elevated expression in PCOS MII oocytes compared to controls (*p* < 0.05, Figure 6A), consistent with its RNA-seq trajectory as a “failed clearance” transcript that abnormally accumulates during maturation. Similarly, *Cox6a1*, a cytochrome c oxidase subunit predicted by trajectory analysis to undergo physiological downregulation during the GV-to-MII transition, instead exhibited a pronounced and significant accumulation in PCOS MII oocytes (RQ = 4.05 vs. 0.30 in controls; *p* < 0.001, Figure 6B), directly corroborating the ‘failed maternal mRNA clearance’ phenotype identified by RNA-seq.

In contrast, *Lsm14b*—the central P-body assembly factor identified as a key regulator of the mRNA decay machinery—demonstrated the opposing “impaired activation” pattern: while control MII oocytes showed a robust ∼9.6-fold upregulation relative to GV-stage controls, PCOS MII oocytes failed entirely to execute this activation (RQ = 1.49 vs. 9.63 in controls; *p* < 0.01, Figure 6C). This blunted activation of *Lsm14b* at the mRNA level provides direct mechanistic support for the hypothesis that defective P-body assembly underlies the global maternal mRNA clearance failure. Finally, *Figla,* an essential transcription factor for oocyte identity maintenance, was dramatically depleted in PCOS MII oocytes (RQ = 0.007 vs. 0.238 in controls; *p* < 0.001, Figure 6D), representing a ∼34-fold greater reduction compared to the already physiologically declining control MII levels.

To validate the transcriptomic findings at the protein level and directly address the inferential gap between mRNA abundance and protein output, we performed Western blot analysis of Lsm14b, Sdhb, and Figla in GV and MII oocytes from control and PCOS mice (150 oocytes per lane, n = 3 biological replicates; Figures 6E,F). Consistent with the RNA-seq and RT-qPCR data, Lsm14b protein was significantly reduced in PCOS MII oocytes compared to controls (0.33 ± 0.01 vs. 1.22 ± 0.02, 3.7-fold reduction, *p* < 0.001), while GV-stage levels showed no significant difference (*p* = 0.644), confirming that the stage-specific activation failure is most pronounced at the MII stage. Sdhb protein was aberrantly elevated in PCOS MII oocytes relative to controls (1.14 ± 0.01 vs. 0.40 ± 0.03, 2.9-fold elevation, *p* < 0.001), demonstrating that the transcript-level retention of this OXPHOS component translates to measurable protein accumulation. Figla protein was markedly depleted in PCOS MII oocytes (0.42 ± 0.03 vs. 0.89 ± 0.03, 2.1-fold reduction, *p* < 0.001), establishing that the ∼34-fold mRNA reduction results in genuine protein-level compromise of oocyte identity maintenance. Collectively, these protein-level findings corroborate the transcriptomic model and confirm that the stage-specific mRNA dysregulation identified in PCOS oocytes has functional consequences at the protein level.

Based on these integrated transcriptomic and experimental findings, we propose a working model for PCOS-induced oocyte decline (Figure 6G). While PCOS oocytes maintain a deceptive baseline stability at the GV stage, their inability to upregulate Lsm14b-mediated maternal mRNA remodeling machinery during the GV-to-MII transition leads to a dual-layered transcriptomic collapse: the pathological retention of metabolic transcripts and the aberrant hyper-depletion of identity networks. This stalled maturation program is characterized by the paradoxical co-occurrence of metabolic transcript retention and identity gene depletion. Together, these dual disruptions establish a transcriptomic signature indicative of OXPHOS hyperactivation, which ultimately converges on a terminal decline in oocyte developmental competence.

## 4 Discussion

The present study provides a comprehensive transcriptomic map of oocyte maturation failure in PCOS, revealing that the pathological impact of PCOS is not a static baseline defect but rather a stage-specific collapse of the post-transcriptional remodeling program that is indispensable for the GV-to-MII transition (Wang et al., 2026; Liu et al., 2016). By integrating multi-layered computational analyses—encompassing differential expression, trajectory modeling, co-expression network construction, and protein–protein interaction mapping—with independent RT-qPCR validation, we demonstrate that PCOS simultaneously disrupts two opposing but complementary processes: the timely clearance of maternal metabolic transcripts, and the preservation of oocyte identity gene networks. The convergence of these two failures on a narrow developmental window defines a previously underappreciated transcriptomic vulnerability that may underlie the poor oocyte competence clinically observed in PCOS patients (Przewocki et al., 2024).

Prior transcriptomic studies of PCOS oocytes have predominantly employed single-timepoint profiling or focused on specific molecular pathways such as mitochondrial dysfunction, oxidative stress, or epigenetic dysregulation (Li et al., 2021; Danfeng et al., 2023; Wang et al., 2024). While these studies have collectively established that PCOS impairs oocyte quality, the precise developmental window at which transcriptomic divergence erupts has remained poorly defined. The present study addresses this gap by performing parallel RNA-seq profiling at both the GV and MII stages, revealing a striking stage-specificity: transcriptomic profiles of control and PCOS oocytes were largely similar at the single-gene level at the GV stage (35 DEGs), with subtle network-level perturbations detectable only by co-expression analysis, yet diverged dramatically upon meiotic maturation (292 DEGs, with the majority of dynamic transcripts diverted into pathological trajectories). This temporal resolution reframes the pathogenesis of PCOS-associated oocyte dysfunction: rather than constituting a pre-existing single-gene lesion that is merely amplified during maturation, the data support a model in which latent, network-level perturbations present at the GV stage are dramatically unmasked and amplified during the GV-to-MII transition, when the oocyte’s capacity for coordinated post-transcriptional remodeling is selectively compromised (Liu et al., 2016). This interpretation is consistent with the clinical observation that PCOS oocytes retrieved at the GV stage appear morphologically normal yet consistently fail to achieve full developmental competence following maturation (Huang et al., 2015).

The present finding of minimal GV-stage transcriptomic divergence (35 DEGs) requires contextualization against published human PCOS oocyte data. Qi et al. (2020) reported premature upregulation of mitochondrial OXPHOS genes (*COX6B1, COX8A, COX4I1, NDUFB9*) at the GV stage in human PCOS oocytes using single-cell RNA-seq. This apparent discrepancy with our data is most likely attributable to species-specific differences between the DHEA-induced hyperandrogenic mouse model and the metabolically heterogeneous human PCOS population, as well as differences in PCOS phenotype composition between cohorts. Consistent with this interpretation, our GSEA analysis confirmed that OXPHOS gene sets were not enriched at the GV stage in our model (NES = +0.095, *p*adj = 0.894), but became significantly enriched at the MII stage (NES = +1.783, *p*adj = 0.002), indicating that the DHEA model captures a maturation-coupled rather than constitutive OXPHOS dysregulation. The DHEA model primarily recapitulates the hyperandrogenic lean PCOS phenotype (Rotterdam phenotypes A/B), whereas human clinical cohorts typically include patients with insulin-resistant and metabolically dysregulated phenotypes in whom constitutive OXPHOS perturbations may manifest earlier. Liu et al. (2016) similarly identified transcriptomic differences across GV, MI, and MII stages in human PCOS oocytes, which is consistent with our observation of 35 GV-stage DEGs—we do not claim transcriptomic identity at the GV stage, but rather that the divergence is quantitatively minimal compared to the dramatic MII-stage collapse (292 DEGs). Taken together, these cross-species comparisons suggest that the precise developmental window of PCOS-induced transcriptomic disruption may vary with PCOS phenotype and model system, and that validation of the Lsm14b–P-body axis in human PCOS oocytes represents an essential next step.

A central mechanistic finding of this study is the failure of Lsm14b to execute its normal MII-stage activation in PCOS oocytes. Lsm14b is a conserved, oocyte-specific RNA-binding protein that functions as a structural scaffold for P-body assembly and mRNA decapping complexes, and whose genetic ablation in mice causes meiotic arrest and female infertility (Wan et al., 2023; Shan et al., 2023). Under physiological conditions, we observed a robust upregulation of *Lsm14b* during the GV-to-MII transition in control oocytes, consistent with the known requirement for P-body remodeling during meiotic maturation (Zhang et al., 2017; Li et al., 2023; Flemr et al., 2010). In PCOS oocytes, this activation was almost entirely abrogated (RT-qPCR: RQ = 1.49 vs. 9.63 in controls; *p* < 0.01), a finding independently confirmed by our transcriptomic trajectory analysis, which classified *Lsm14b* as an “Impaired in PCOS” gene. Critically, the GV-to-MII transition relies heavily on the translation of stored maternal mRNAs rather than *de novo* transcription (Jiang et al., 2023; Sha et al., 2019); therefore, the profound failure to upregulate Lsm14b restricts the molecular stoichiometry required for proper P-body assembly (Brandmann et al., 2018), providing a mechanistically coherent explanation for the global maternal mRNA clearance failure observed in this study. Western blot analysis confirmed that this blunted mRNA activation is mirrored at the protein level, with Lsm14b protein significantly reduced in PCOS MII oocytes compared to controls (3.7-fold reduction**,** *p* < 0.001; Figures 6E,F), establishing that the transcriptional suppression of *Lsm14b* has direct functional consequences for P-body protein stoichiometry. Whether this transcriptional suppression of *Lsm14b* reflects upstream epigenetic silencing, altered transcription factor occupancy, or post-transcriptional destabilization under hyperandrogenic conditions remains an important open question for future investigation (Rambaran and Islam, 2025).

The identification of mitochondrial oxidative phosphorylation (OXPHOS) transcripts—including *Sdhb* and *Cox6a1*—as the dominant class of pathologically retained transcripts in PCOS MII oocytes represents a conceptual departure from prevailing interpretations of mitochondrial dysfunction in PCOS. Previous studies have largely attributed the mitochondrial abnormalities of PCOS oocytes to impaired biogenesis or intrinsic organelle dysfunction (Siemers et al., 2023; Zhang et al., 2019). Our data suggest an alternative, and potentially complementary, mechanism: rather than reflecting newly generated dysfunctional transcripts, the aberrant accumulation of OXPHOS mRNAs in PCOS MII oocytes may represent a failure of the programmed clearance that normally silences these transcripts during the metabolic quiescence required for meiotic completion. This pathological retention of metabolic transcripts may drive untimely translational activity during a developmental stage that demands transcriptomic silence, potentially generating a state of OXPHOS hyperactivation that is incompatible with normal oocyte maturation (Adhikari et al., 2022; Kirillova et al., 2021). Consistent with this hypothesis, Western blot analysis demonstrated that Sdhb protein is aberrantly elevated in PCOS MII oocytes compared to controls (2.9-fold elevation, *p* < 0.001; Figures 6E,F), confirming that the transcript-level retention of this OXPHOS component translates into measurable protein accumulation and supporting the concept of pathological OXPHOS hyperactivation during meiotic maturation.

The extreme depletion of *Figla* in PCOS MII oocytes (RT-qPCR: RQ = 0.007 vs. 0.238 in controls; approximately 34-fold greater reduction) warrants particular attention. Figla is an oocyte-specific basic helix-loop-helix (bHLH) transcription factor that is essential for the transcriptional activation of zona pellucida genes (*Zp1, Zp2, Zp3*) and the maintenance of the oocyte transcriptional identity program (Liang et al., 1997; Soyal et al., 2000; Wang et al., 2020). While a degree of *Figla* mRNA decline is physiologically expected during the GV-to-MII transition as part of the global maternal mRNA clearance program (Wu and Dean, 2020), the near-complete depletion observed in PCOS oocytes represents a pathological amplification of this process that far exceeds the physiological range. This hyper-depletion of *Figla* mRNA may compromise the translational reserve available for zona pellucida protein synthesis and downstream oocyte identity maintenance (Litscher and Wassarman, 2020; Canosa et al., 2017), potentially contributing to the poor fertilization rates and reduced oocyte developmental competence commonly observed in IVF cycles of PCOS patients (Yang et al., 2021). Western blot analysis confirmed that the ∼34-fold mRNA depletion of Figla in PCOS MII oocytes translates directly to a significant reduction in Figla protein (2.1-fold reduction, *p* < 0.001; Figures 6E,F), establishing that the pathological hyper-clearance of *Figla* mRNA results in genuine protein-level compromise of oocyte identity maintenance and providing direct evidence that the transcriptomic disruption observed in PCOS oocytes has functional consequences for the zona pellucida protein synthesis machinery.

Integrating these findings, we propose a working model in which the failure of Lsm14b -mediated maternal mRNA remodeling machinery serves as a proximal mechanistic node linking the hyperandrogenic PCOS microenvironment to the dual-layered transcriptomic collapse observed at the MII stage. In this model, the inability to upregulate *Lsm14b* and its co-regulated P-body components during the GV-to-MII transition impairs the assembly of functional RNA decay complexes (Li et al., 2023; Wan et al., 2023), resulting in the simultaneous pathological retention of metabolic transcripts (*Sdhb, Cox6a1*) and the aberrant hyper-depletion of oocyte identity factors (*Figla*). This paradoxical dichotomy—wherein the collapse of Lsm14b-mediated coordinated decay stalls the clearance of metabolic targets while failing to protect identity transcripts—suggests that the disruption of P-body stoichiometry does not simply silence all decay activity uniformly, but rather destabilizes the global RNA homeostatic network (Shan et al., 2023). To provide bioinformatic support for this differential pathway model, we performed a transcriptome-wide analysis of 3′UTR sequence features distinguishing the two transcript classes. Retained transcripts (failed clearance class; n = 354 genes) were significantly enriched for Pumilio response elements (PRE; TGTA [N]ATA motifs) compared to hyper-depleted transcripts (n = 419 genes; 10.6% vs. 7.0% of transcript sequences, Fisher’s OR = 1.57, *p* = 0.0004). PREs are canonical binding sites for Pumilio family proteins that cooperate with Lsm14b and P-body components to mediate ARE-dependent mRNA decay, consistent with the retained transcripts being primary substrates of the Lsm14b -dependent decay machinery. Conversely, hyper-depleted transcripts exhibited significantly higher 3′UTR GC content (median 0.473 vs. 0.452, Wilcoxon *p* < 0.001), a sequence feature associated with codon-optimized transcripts that are preferentially targeted by the CCR4-NOT deadenylation complex through codon-usage-dependent mechanisms (Liu et al., 2024). These findings suggest that the two transcript classes are subject to distinct decay machineries: retained OXPHOS transcripts are canonical Lsm14b/P-body substrates whose clearance is directly impaired by Lsm14b failure, while identity transcripts are preferentially targeted by Lsm14b -independent CCR4-NOT-mediated deadenylation that proceeds—and may even accelerate—in the absence of coordinated P-body activity. The net consequence is a transcriptomic landscape that is neither properly cleared nor properly maintained—a state of coordinated post-transcriptional dysregulation that is incompatible with the acquisition of full developmental competence. While our model positions the failure of Lsm14b -mediated decay as a proximal driver of oocyte decline, the precise upstream epigenetic or signaling mediators linking the hyperandrogenic microenvironment to the suppression of *Lsm14b* activation remain a critical missing piece of the puzzle, warranting future multi-omics investigations targeting the androgen receptor signaling axis and its downstream transcriptional consequences in the oocyte nucleus (Rambaran and Islam, 2025).

Several limitations of the present study merit consideration. First, the DHEA-induced mouse model primarily recapitulates the hyperandrogenic lean phenotype of PCOS (Rotterdam phenotypes A/B) and may not fully capture the metabolic and endocrine heterogeneity of human PCOS, particularly the insulin-resistant subtype. Whether the transcriptomic disruptions identified here are conserved across different PCOS etiologies—including letrozole-induced or prenatal androgenization models—and in human oocytes remains to be established; validation in human PCOS oocyte cohorts is an essential next step. Second, the mechanistic framework proposed in this study is constructed entirely at the transcriptomic level. Direct evidence for the downstream consequences of *Lsm14b* failure—including Lsm14b protein abundance, P-body physical assembly defects (e.g., DDX6/EDC4 immunofluorescence foci), altered decapping activity, and protein-level dysregulation of Figla and OXPHOS components—awaits validation by single-cell proteomics and fluorescence-based P-body imaging approaches (Sun et al., 2023; Swetloff et al., 2009). Similarly, direct assessment of oocyte quality endpoints—including spindle assembly integrity, mitochondrial membrane potential, ROS levels, and fertilization competence—was not performed in this study; the molecular evidence presented here requires correlation with these functional quality metrics in future work. Third, the correlative nature of the transcriptomic data precludes definitive causal inference. Establishing whether *Lsm14b* activation failure is causally sufficient to recapitulate the observed clearance defects will require functional intervention experiments, such as microinjection-based *Lsm14b* knockdown or mRNA rescue in in vitro maturation (IVM) models (Gong et al., 2023). Notably, Li et al. (2024) demonstrated that restoration of Lsm14b function in obese mice improved oocyte quality and rescued maternal mRNA storage defects, providing indirect support for the causal role of Lsm14b in oocyte competence and establishing a precedent for IVM-based rescue experiments as a priority for future investigation.

The findings of this study carry potential translational implications for the clinical management of PCOS-associated infertility. The following implications are proposed as hypotheses for future investigation, rather than conclusions directly supported by the present transcriptomic data. Specifically, recognizing the GV-to-MII transition as a discrete transcriptomic vulnerability window suggests that metabolic and post-transcriptional interventions could complement traditional systemic hormonal therapies (Gotschel et al., 2024). This is particularly relevant for IVM protocols, which are increasingly employed in PCOS patients to reduce the risk of ovarian hyperstimulation syndrome; the present findings suggest that the molecular competence of the GV-to-MII transition—rather than the hormonal milieu alone—should be considered a primary optimization target in IVM culture system design (Gargallo-Alonso et al., 2026; Moreira et al., 2023). Supplementing IVM media with agents that promote P-body assembly or restore coordinated maternal mRNA remodeling could potentially bypass this stage-specific molecular roadblock (Li et al., 2024). In particular, the Lsm14b–P-body axis emerges as a candidate molecular target whose activation state may serve as a functional indicator of oocyte maturation competence. Furthermore, the dual-layered transcriptomic signature identified here—characterized by the co-occurrence of metabolic transcript retention and identity gene depletion—may provide a molecular basis for developing non-invasive biomarkers of oocyte quality in clinical IVF settings (Daugelaite et al., 2025), pending validation in human oocyte cohorts. Future studies should prioritize the characterization of the upstream regulatory network governing Lsm14b activation in the oocyte, the development of IVM-based functional rescue models, and the cross-species validation of these transcriptomic signatures in human PCOS oocytes.

In summary, this study redefines PCOS-associated oocyte incompetence not as a constitutive baseline lesion, but as a stage-specific failure of post-transcriptional remodeling that erupts exclusively during the GV-to-MII transition. The blunted activation of *Lsm14b* emerges as a proximal mechanistic node, precipitating a paradoxical dual-layered transcriptomic collapse characterized by the pathological retention of metabolic transcripts (*Sdhb, Cox6a1*)—indicative of untimely OXPHOS hyperactivation—alongside the aberrant hyper-depletion of essential oocyte identity genes (*Figla, Zp3*). By positioning the Lsm14b–P-body axis at the intersection of maternal mRNA clearance and oocyte identity maintenance, our findings provide a mechanistically coherent framework for understanding the qualitative oocyte defect in PCOS and identify a candidate molecular target for the rational optimization of *in vitro* maturation protocols and the development of non-invasive oocyte quality biomarkers in clinical assisted reproduction.

## Data Availability

The datasets presented in this study can be found in the NCBI Gene Expression Omnibus (GEO) repository under accession number GSE328232 (https://www.ncbi.nlm.nih.gov/geo/query/acc.cgi?acc=GSE328232).

## Supplementary material

SUPPLEMENTARY FIGURE S1 RNA-seq quality control and outlier sensitivity analysis. **(A)** Library size distribution across all 14 samples before and after normalization. **(B)** Expression distribution (violin plots) of log_2_-normalized counts across all samples. **(C)** Sample correlation heatmap showing high intra-group reproducibility. **(D)** Principal component analysis (PCA) of all 14 samples (left panel), showing GV_Ctrl_4 as a clear outlier deviating >3 standard deviations from the GV Control group centroid in the full 4-group PCA space; and PCA of the final 13-sample dataset after outlier exclusion (right panel). **(E)** Sensitivity analysis: differential expression analysis at the GV stage retaining GV_Ctrl_4 (n = 4 GV Control samples) yielded 33 DEGs, compared to 35 DEGs in the primary analysis (n = 3 GV Control samples), confirming that the central conclusion of minimal GV-stage transcriptomic divergence is robust to outlier inclusion. None of the key hub genes (*Lsm14b, Sdhb, Cox6a1, Figla, Zp3*) were differentially expressed at the GV stage in either analysis.
